# Statistical Approaches Based on Deep Learning Regression for Verification of Normality of Blood Pressure Estimates

**DOI:** 10.3390/s19092137

**Published:** 2019-05-08

**Authors:** Soojeong Lee, Gangseong Lee, Gwanggil Jeon

**Affiliations:** 1 Department of Computer Engineering, Sejong University, 209 Neungdong-ro, Gwangjin-gu, Seoul 05006, Korea; leesoo86@sejong.ac.kr; 2 Ingenium College, Kwangwoon University, 20 Kwangwoon-ro, Nowon-gu, Seoul 01897, Korea; gslee0115@gmail.com; 3 School of Electronic Engineering, Xidian Unversity, No. 2 South Taibai Road, Xi’an 710071, China; 4 Department of Embedded Systems Engineering, Incheon National University, 119 Academy-ro, Yeonsu-gu, Incheon 22012, Korea

**Keywords:** blood pressure, oscillometric measurement, statistical analysis, normality, confidence limit, deep learning

## Abstract

Oscillometric blood pressure (BP) monitors currently estimate a single point but do not identify variations in response to physiological characteristics. In this paper, to analyze BP’s normality based on oscillometric measurements, we use statistical approaches including kurtosis, skewness, Kolmogorov-Smirnov, and correlation tests. Then, to mitigate uncertainties, we use a deep learning method to determine the confidence limits (CLs) of BP measurements based on their normality. The proposed deep learning regression model decreases the standard deviation of error (SDE) of the mean error and the mean absolute error and reduces the uncertainties of the CLs and SDEs of the proposed technique. We validate the normality of the distribution of the BP estimation which fits the standard normal distribution very well. We use a rank test in the deep learning technique to demonstrate the independence of the artificial systolic BP and diastolic BP estimations. We perform statistical tests to verify the normality of the BP measurements for individual subjects. The proposed methodology provides accurate BP estimations and reduces the uncertainties associated with the CLs and SDEs using the deep learning algorithm.

## 1. Introduction

Blood pressure (BP) is a key consideration when making decisions about the cardiovascular activity of patients. The oscillometric method has lately been popularly and is utilized in automatic BP measurement devices that are now readily accessible in the marketplace. Though oscillometric BP device’s manufacturers rarely disclose algorithms, but the literature mentions that the maximum amplitude technique is most commonly used to estimate arterial blood pressure (ABP) [1]. The mean arterial BP is easily estimated via the cuff pressure whereby the oscillation amplitude becomes maximal utilizing the oscillometric waveform when the cuff pressure matches the average arterial BP. The compliance of the arterial wall is then maximized, so the ABP volume change with respect to the maximum change in the arterial pressure and the amplitude of the oscillation signal in the cuff is also at maximum. Even though the ABP estimation relies on the oscillometric technique, it causes a variety of errors because the BP signals are characterized by continuous variations. Specifically, the systolic BP (SBP) and diastolic BP (DBP) are subject to significant and continuous change over time [2] in response to physiological oscillations due to factors such as food intake, emotional status, level of exercise, and disease states, such that they can fluctuate to 20 mmHg for several heartbeats [3]. However, the SBP and DBP are generally estimated at two random instances in time with respect to the systolic and diastolic ratios (SBPR and DBPR) in the oscillometric waveform envelope [2], which offers no information on the significant BP fluctuations. There is no established criterion for determining accurate SBP and DBP or SBPR and DBPR [4]. A journal editorial asserted that there is an acutely aware of biological fluctuations [5]. The element of accurate BP estimation follows: the first is the accuracy of the measurement system and the second is the physiological variability. The first source of uncertainty is represented by BP measurement error according to the ANSI/AAMI SP 10 standard [6], in which physiological variations of the BP are overlooked by the great majority physicians. Therefore, oscillometric BP devices generally provide a single parameter with no confidence limit (CL) to distinguish them from the fluctuations of physiological characteristics [5]. If CL estimates are applied to the BP monitor, the results of the wide CL estimate on the BP value can be determined by the unstable BP value. Conversely, a narrow CL estimate can be determined with a stable BP value. However, since repeatable status to recreate measurements cannot be assured, it is practically and economically impossible to acquire lots of measurements to one subject utilizing an oscillometric-based BP monitor [1]. In such a real situation, CL should be estimated using a small number of BP values measured over a short time. Thus, using the bootstrap technique, a CL estimate can be acquired using a BP value of a small sample size [1]. Unfortunately, this approach assumes that BP based on the oscillometric measurement for individual subjects is an independent and identical distribution (iid), which supposes that the populations for which BP are measured are normally distributed. The assumption of normality is especially critical when determining CLs for BP measurement for individual subjects. The normality assumption is a key factor in the reliability of estimations and statistical tests [7]. Thus, this assumption should be taken seriously because it is impossible to estimate BP accurately when this assumption does not hold. For this reason, the assumption of normality should be verified with respect to BP measurements for individual subjects.

In this investigation, our research objective is to use statistical approaches including kurtosis, skewness, Kolmogorov-Smirnov (KS), and rank tests to examine the normality of BP values based on oscillometric measurements. We also estimate the SBP, DBP, and CLs of these values based on the normality for individual subject using a deep learning [8]. Deep learning is one part of machine learning techniques that models high-level abstractions in data by utilizing multi-layered architecture with complex nonlinear transformations [9]. However, our BP measurements for individual subjects are drawn from only a small sample size due to limited measurements available for individual subjects, which is a disadvantage when using a deep learning that works best with big data [10]. To address this problem, we first generate artificial features from the original data using a parametric bootstrap technique for the SBP and DBP estimates. This approach can efficiently represent a very complex relationship between the feature data acquired from oscillometric signals and those from target BPs. Then, we utilize the bootstrap technique again to decide the CLs using the estimated target BP for the deep learning technique. Next, we statistically analyze the normality of the BP measurements. We believe this to be one of the first studies using statistical analysis for individual BP estimation as the contributions below:

- We propose a new approach for estimating BP parameters that mitigates uncertainties (physiological variability), such as the CLs and standard deviation of errors, using the deep learning technique. To do so, we use a small sample of oscillometric BP measurements and bootstrap techniques for individual subjects.
- First, we perform the kurtosis and skewness tests to verify the normality of the BP measurements for individual subjects.
- We then use a rank test to analyze the independence between the artificial BPs estimations.

**Figure 1:**
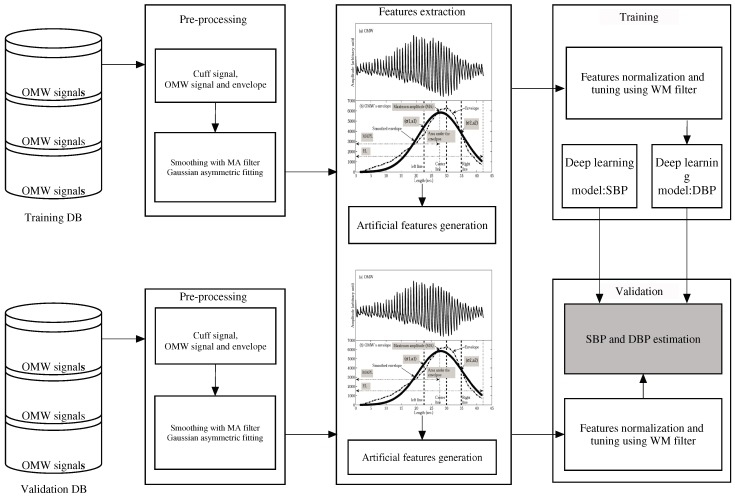
Flow chart of the proposed technique.

Figure 1 shows the introduced methodology and we describe the procedure below. The upper part in the figure shows the first procedure in which the proposed algorithm is trained, and the lower part illustrates the validation procedure. We combine these two parts to estimate SBP and DBP. After bio-signal processing on the BP signals, we obtain the input data from the oscillometric waveform (OMW) signals and envelopes. We then perform the pre-processing as shown in Figure 1. Next, we generate the artificial features from the original features and evaluate the normality of the distribution of all the features. We then build the deep learning model via the two training processes and perform pre-training and fine-tuning using a back-propagation algorithm [8,10]. In the second procedure, we use the unseen data set to evaluate the proposed methodology. Using the deep learning regression model, we estimate the target BP values (SBP and DBP) for the individual subjects. Subsequently, to determine the CLs of the BPs, we generate artificial BPs using target BP values generated using the parametric bootstrap scheme. Finally, we fully verify the normality of the artificial BPs for individual subjects.

In the next Section 2, we explain the experimental data and BP measurement procedures. In Section 3, we briefly describe the oscillometric BP estimation using the deep learning regression model. We describe the statistical analysis of the BP estimation and verify the normality of BP values based on oscillometric measurements in Section 4. In Section 5, we present the results and conclusions.

## 2. Data Set

This research has been passed by a research ethics committee and all test volunteers agreed before the BP measurement. The experimental BP data were originally recorded from 85 healthy people with free from cardiovascular disease, age from 12 to 80, 48 man and 37 women as shown in Table 1. We obtained five sets of oscillometric BP measurements from each volunteer using a wrist-mounted blood pressure monitor with the ANSI/AAMI standard protocol [6,11]. We averaged the readings made by two independent specialists (nurses) to yield the SBP and the DBP target value [1]. This procedure was repeated four more times to record five measurements. There was a one-minute break between each measurement. Each volunteer sat comfortably in a chair during BP measurements, which wound the BP cuff around the volunteer’s left wrist and could be raised to a heart level. The reference device, auscultatory BP cuff, was wound around the upper left arm to match the heart level. The upper left cuff was bloated around the arm so as to occlude the brachial artery. When the upper cuff was deflated, the blood flow produced Korotkoff (K) signals that could be heard with a stethoscope placed beside the upper left cuff. The first K signal (K1) that was measured in mmHg by a manometer of the upper left cuff, was used to predict SBP, while the fifth signal (K5) was utilized to predict DBP [11]. It was not possible to measure the upper left arm and wrist BP measurements at the same time due to the difficulty of occlusion of brachial arteries through upper left arm sphygmomanometers. Thus, nearly 1.5 (min.) after each signal was acqured by the device of the wrist measurement, two medical staffs concurrently measured SBP and DBP utilizing a sphygmomanometer.

In our procedure, we sequentially separated the BP data of the voluntary workers into a learning set (300 measurements obtained from 60 volunteers with five data sets) and validation set (125 data acquired from 25 volunteers with five data sets). Then, we repeated this procedure to ensure that individual volunteers were included only once in the validation procedure. Five BP data in an individual volunteer constitute only a small number of input data in the learning procedure. Thus, we also utilized the artificial data generated from the real data. We used the unseen data to evaluate our methodology. As mentioned above, we obtained our data from the oscillometric waveforms, which we utilized to create artificial data based on the parameter bootstrap technique [12].

## 3. Deep Learning-Based Regression Estimator

### 3.1. Features Obtained from Oscillometric Signals

We extract informative features from the oscillometric waveform signals [4] and build envelopes after processing the signals of the oscillometric BP measurements for estimating the reference BP values. We then utilize these estimates as the reference BPs in the proposed deep learning algorithm. More details regarding these features can be found in [4] for the interested readers.

### 3.2. Artificial Data Obtained Using Bootstrap

Since we have only five BP measurements for individual subjects, we create artificial input data utilizing the bootstrap technique as in [1,12], which is a technique for improving the exactness of estimates using a limited data set for situations in which traditional approaches are ineffective for use in enhancing the exactness [1,12]. Here, we assume $X=[x_{1},\ldots ,x_{N}]$ to be a random data of the distribution T with unknown values [θ,σ]. We then approximate T by $\hat{T}\left(\hat{\theta },\hat{\sigma }|X\right)$, where we define the average and standard deviation by $E\left(\theta |X\right)≃\hat{\theta }=\bar{x}=\frac{1}{N}\sum_{n=1}^{N}x_{i}$ and $E\left(\sigma |X\right)≃\hat{\sigma }=\sqrt{\left[\frac{1}{N-1}\sum_{n=1}^{N}{\left(x_{n}-\bar{x}\right)}^{2}\right]}$, respectively, where $\hat{T}≃N\left(\hat{\theta },\hat{\sigma }\right)$ is approximated by a Gaussian distribution, that is known as the parametric bootstrap technique [12]. We utilize this parametric way to create the artificial features ${\hat{\Xi }}^{*}=\left[\theta_{1}^{*},\theta_{2}^{*},\ldots ,\theta_{B}^{*}\right]$, which we acquire from the real input data X.

### 3.3. KS Analysis for Data

**Figure 2:**
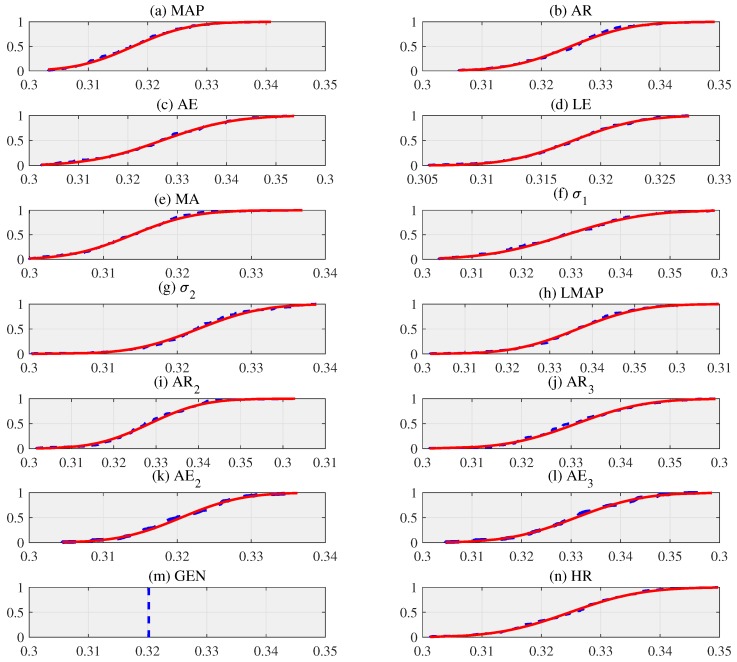
The cumulative distribution function (CDF) of artificial input data using the bootstrap technique with replication size (*N* = 100), where artificial input data are examples acquired from a volunteer with 5 samples [11,14].

To use the artificial feature distribution with confidence, we fully evaluate the normality of each feature. First, we conduct the KS test to verify the normality of each artificial data distribution. Thus, we suppose that $T^{*}$ is a distribution of an artificial input data $\left[\theta_{1}^{*},\theta_{2}^{*},\ldots ,\theta_{N}^{*}\right]$, where *N* is the size of replication. We present the probability of measuring the equality between the distribution of artificial input data and hypothesis [11]. Based on the test results, we clearly confirm the distribution of the artificial feature to be a cumulative distribution function (CDF) as shown in Figure 2, which shows the plots of the distribution of artificial features that fit the normal distribution. As the size of *N* replication increases, the distribution more closely adheres to a normal distribution [1]. If the results of the every artificial input data in KS test set to 0, as represented in Table 2, we can not reject the null hypothesis at the α (=0.05) significance level. Moreover, we note that all *p* values of the KS test are greater than the α (=0.05) significance level. In addition, if the KS test values ks are greater than the critical values cv, the null hypothesis will be rejected. Thus, we accept the null hypothesis that distributions of the artificial features follow Gaussian distributions [13].

We also validate the consistency and convergence of the artificial features obtained by bootstrap method [12]. Thus, our artificial features should fit well with real features using the bootstrap convergence for the sample mean based on the theorem [15] as

**Theorem** **1.** *If $E(x^{2})<\infty$, then*

(1) $$\|T^{*}\left\{\sqrt{n}\left({\hat{\theta }}^{*}-\hat{\theta }\right)\leq x\right\}-T\left\{\sqrt{n}\left(\hat{\theta }-\theta \right)\leq x\right\}{\|}_{\infty }⟶0$$

where $T^{*}$ represents the conditional distribution based on the bootstrap technique using the real feature X and ${\left\|\cdot \right\|}_{\infty }$ is $\sup_{x\in R}\left|\cdot \right|$.

Therefore, it can be confirm that the distribution of $\sqrt{n}\left({\hat{\theta }}^{*}-\hat{\theta }\right)$ is close to $\sqrt{n}\left(\hat{\theta }-\theta \right)$ [15].

(2) $$\beta \left(\theta \right)=E\left(\hat{\theta }\left(X\right)-\theta )\right)$$

where β is a bias and θ is the real feature. If the bias is close to zero, the estimate is considered to be almostly unbiased. Thus, we calculate the bias and standard error of the artificial feature as follows:

(3) $$\begin{array}{cc} \beta ({\hat{\theta }}^{*}\left(\cdot \right))= & \frac{1}{N}\sum_{b=1}^{N}{\hat{\theta }}_{b}^{*}-E\left(\theta |X\right)\\ = & E\left(\theta |X^{*}\right)-E\left(\theta |X\right)\\ = & {\hat{\theta }}^{*}-E\left(\theta |X\right)\\ ≅ & E\left({\hat{\theta }}^{*}\left(X^{*}\right)-\hat{\theta }\left(X\right)\right) \end{array}$$

where $\beta ({\hat{\theta }}^{*}\left(\cdot \right))$ is the prediction error (i.e., bias).

(4) $${\hat{S}}_{e}^{*}=\sqrt{\left(\frac{1}{N-1}\sum_{b=1}^{N}{\left({\hat{\theta }}^{*}-{\hat{\theta }}^{*}\left(\cdot \right)\right)}^{2}\right)}$$

where ${\hat{S}}_{e}^{*}$ represents the standard error using the parametric bootstrap and ${\hat{\theta }}^{*}\left(\cdot \right)$ is $N^{-1}\sum_{b=1}^{N}{\hat{\theta }}_{b}^{*}$. We find that the bias of the artificial features are very small in the exemplary sample and that the standard error ${\hat{S_{e}}}^{*}$ acquired from the bootstrap technique more closely approximates $\hat{\theta }$ than $\hat{S_{e}}=\hat{\sigma }$, thus the bootstrap technique can be utilized as a good producer for increasing the number of samples for the artificial features as shown in Table 3. Therefore, The artificial features are found to be very close to the real features. We also find that the CLs of the artificial features include all artificial and real features as shown in Table 3. The remaining features represent very similar results as shown in Table 3.

### 3.4. Deep Learning Based Regression

We can see that the deep learning estimator is essentially based on the distributed representation, which implies that we can describe the obtained data by the interactions of various components at different levels [9]. We organize our deep learning estimator in two training procedures, with a pre-learning and tuning with respect to the target BPs. The pre-learning phase consists of deep learning, which calculate the highest layer to the lowest layer. The deep learning is a probability generation model that consists of several hidden layers. The top two layers consist of non-directional connections, whereas the hidden layers compose a top-down acyclic structure, where the units in the lower layer represents the input vectors, which are subsequently connected to two layers known as the restricted Boltzmann machine (RBM) [8]. The RBM is a basic component of the deep learning that contains several hidden layers. Our model of deep learning is given as $P\left(X^{*},s^{1},s^{2},\ldots ,s^{l}\right)=P\left(X^{*}|s^{1}\right)P\left(s^{1}|s^{2}\right)\cdots P\left(s^{l-2}|s^{l-1}\right)P\left(s^{l-1},s^{l}\right)$, where the probabilities of the condition layers $P(s^{i}|s^{i+1})$, $s^{i}$ represent the hidden units at layer *i*, and $X^{*}$ denotes the re-sampled input data. We can define a probability as follow:

(5) $$P\left(X^{*},s\right)=\frac{1}{Q}\exp^{-s^{\prime }WX^{*}-c^{T}X^{*}-b^{T}s}$$

where *Q* denotes the partition function, c denotes the bias of the input data, b is the bias of the hidden units, and W is the weight values. Thus, we can define the conditional probability of a layer, given the other by $P\left(s^{i}|s^{i+1}\right)=\mathrm{sigm}\left(-c-\sum_{k=1}^{n^{i+1}}Ws_{k}^{i+1}\right)$, where sigm(x) is 1/(1+exp(−x)), $c=[c_{j}^{i}]$ and denotes the bias for unit *j* of layer *i*, and $W=[W_{kj}^{i}]$ is the weight matrix for layer *i*. To connect an input layer with a hidden layer, we use the Gaussian-Bernoulli RBM (GBRBM) [8], because we assume the artificial input data to follow an asymptotic normal distribution. Then, we stack multiple Bernoulli-Bernoulli RBMs (BBRBMs) behind the first GBRBM [8]. Next, we train the second BBRBM by using the hidden layer of the first GBRBM [8]. In the learning procedure, we use pre-learning to initialize weights and biases and use them like an efficient starting point [9] to fine-tune by stochastic gradient descient. We use the minimum mean squared error function to estimate the cost function [16] as follows:

(6) $$\Omega \left(W,c\right)=\frac{1}{B}\sum_{n=1}^{B}\sum_{d=1}^{D}{\left({\hat{Y}}_{n}^{*d}\left(W,c\right)-Y_{n}^{d}\right)}^{2}$$

where Ω is a loss function; ${\hat{Y}}_{n}^{*d}\left(W,c\right)$ and $Y_{n}^{d}$ denote the *d*th hypothesis and target BP data at the sample index *n*, respectively; *D* and *B* represent the size of data and the size of batch, respectively; and $\left(W^{i},c^{i}\right)$ is the weight and bias values learned at the *i*th layer. Then, we can iteratively update the estimated weights and bias, as follows:

(7) $$\left(W_{n+1}^{i},c_{n+1}^{i}\right)=-\epsilon \frac{\partial \Omega }{\partial \left(W_{n}^{i},c_{n}^{i}\right)}+\eta \left(W_{n}^{i},b_{n}^{i}\right),1\leq i\leq H+1$$

where ϵ is a learning rate, η denotes a momentum value, *H* is the size of hidden layers, and H+1 is the output layer.

## 4. Statistical Analysis for BP Estimation

### 4.1. CL Estimation

Our goal in estimating the CLs is to utilize the bootstrap algorithm to identify the uncertainty (physiological variability) and to provide the CLs of five BP estimations obtained from the deep learning regression model for individual subjects. First, we describe the bootstrap principles of the nonparametric and parametric approaches. The basic idea is to generate many artificial BP estimates by resampling real BP estimates, $X=[x_{1},\ldots ,x_{n}]$, utilizing *n* independent measurements from an unknown probability distribution T to generate a CL for $\hat{\mu }\left(X\right)$. In addition, we assume X to be a random data of the distribution T with unknown parameter [μ,σ]. As such, we sample the artificial sample $X^{*}$ from $\hat{T}\left(\hat{\mu },\hat{\sigma }|X\right)$ using the Monte-Carlo approach, whereby $\left[\hat{\mu },\hat{\sigma }\right]$ is generally the maximum likelihood estimate from $X=[x_{1},\ldots ,x_{n}]$. Therefore, when N→∞, we can approximate a Gaussian distribution as given by $\hat{F}\left({\hat{\mu }}^{*},{\hat{\sigma }}^{*}|X^{*}\right)≅N\left(\mu ,\sigma \right)$.

In our approach, we an also obtain the CLs using the bootstrap technique, which was estimated utilizing the BP target values in the deep learning technique. First, the estimated BP $\left(Y^{*}\right)$ values is as given in line 1 of the Algorithm 1. We then acquire two matrices as given by the following:

(8) $$M^{S*}\left(i|{\hat{Y}}_{i}^{S*}\right)=\left[{\left[s_{i,b}^{j}\right]}^{T},\ldots ,{\left[s_{i,b}^{j}\right]}^{T}\right]$$

(9) $$M^{D*}\left(i|{\hat{Y}}_{i}^{D*}\right)=\left[{\left[d_{i,b}^{j}\right]}^{T},\ldots ,{\left[d_{i,b}^{j}\right]}^{T}\right]$$

where Equations (8) and (9) obtain using the lines 4 and 5 of below algorithm, here, S and D indicate SBP and DBP, respectively, and * denotes the resampled data from the bootstrap technique. We then vertically calculate each column, as lines 7 and 8 of the algorithm, to acquire the average of each column. We then perform ascending sorts as shown in lines 10 and 11. The sorted values are given as ${\hat{\Xi }}^{S*}=\left[{\hat{\theta }}_{1}^{S*},{\hat{\theta }}_{2}^{S*},\cdots ,{\hat{\theta }}_{N}^{S*}\right]$, supposing ${\hat{\theta }}_{\alpha }^{S*}$ is the 100α th percentile of *N* bootstrap replications $\left[{\hat{\theta }}_{1}^{S*},{\hat{\theta }}_{2}^{S*},\cdots ,{\hat{\theta }}_{N}^{S*}\right]$. We obtain the percentile interval ${\hat{\theta }}_{\mathrm{lower}}^{S*},{\hat{\theta }}_{\mathrm{upper}}^{S*}$ of the 1−2·α, from this bootstrap technique, as follows:

(10) $$\left[{\hat{\theta }}_{\mathrm{lower}}^{S*},{\hat{\theta }}_{\mathrm{upper}}^{S*}\right]=\left[{\hat{\theta }}_{\alpha }^{S*},{\hat{\theta }}_{1-\alpha }^{S*}\right].$$

**Algorithm 1:** CL using bootstrap based on deep learning.Procedure CL (${\hat{Y}}^{*}$) 1. For i←1,B${\hat{Y}}_{i}^{{\;}_{S}}=\left\{{\hat{s}}_{1},...,{\hat{s}}_{n}\right\}$ and ${\hat{Y}}_{i}^{{\;}_{D}}=\left\{{\hat{d}}_{1},...,{\hat{d}}_{n}\right\}$ 2. ${\hat{\mu }}_{i}^{{\;}_{S}}=\frac{1}{n}\sum_{j=1}^{n}{\hat{s}}_{j}$ and ${\hat{\mu }}_{i}^{{\;}_{D}}=\frac{1}{n}\sum_{j=1}^{n}{\hat{d}}_{j}$ 3. $E({\hat{\mu }}_{i}^{{\;}_{S}},{\hat{\sigma }}_{i}^{{\;}_{S}}|{\hat{Y}}_{i}^{{\;}_{S}})$ and $E({\hat{\mu }}_{i}^{{\;}_{D}},{\hat{\sigma }}_{i}^{{\;}_{D}}|{\hat{Y}}_{i}^{{\;}_{D}})$ 4. $M^{{\;}_{S*}}\left(i|{\hat{Y}}_{i}^{{\;}_{S}}\right)$ = ${\hat{\mu }}_{i}^{{\;}_{S}}+{\hat{\sigma }}_{i}^{{\;}_{S}}\times \mathrm{RANDN}\left(n,N\right)$ 5. $M^{{\;}_{D*}}\left(i|{\hat{Y}}_{i}^{{\;}_{D}}\right)$ = ${\hat{\mu }}_{i}^{{\;}_{D}}+{\hat{\sigma }}_{i}^{{\;}_{D}}\times \mathrm{RANDN}\left(n,N\right)$ 6. For b←1,N do 7. ${\hat{\theta }}^{{\;}_{S*}}\left(i,b|{\hat{Y}}_{i}^{{\;}_{S}}\right)$ = $\frac{1}{n}$$\sum_{j=1}^{n}$$s_{i,b}^{j}$ 8. ${\hat{\theta }}^{{\;}_{D*}}\left(i,b|{\hat{Y}}_{i}^{{\;}_{D}}\right)$ = $\frac{1}{n}$
$\sum_{j=1}^{n}$$d_{i,b}^{j}$ 9. End For 10. ${\hat{\Xi }}_{i}^{{\;}_{S*}}$ = SORT
$\left[{\hat{\theta }}^{{\;}_{S*}}\left(i,b|{\hat{Y}}_{i}^{{\;}_{S}}\right)\right]$ 11. ${\hat{\Xi }}_{i}^{{\;}_{D*}}$ = SORT
$\left[{\hat{\theta }}^{{\;}_{D*}}\left(i,b|{\hat{Y}}_{i}^{{\;}_{D}}\right)\right]$ 12. End For End Procedure

Where *B* is the number of volunteers, and *n* and *N* denote the number of measurements and replications, respectively, for individual subjects. Due to the physiological characteristics of individual subjects and the cost of the experiment, it is difficult to obtain many BP measurements. Therefore, we estimate CLs utlizing the bootstrap method based on the deep learning algorithm.

### 4.2. Computing and Testing for Kurtosis and Skewness

To verify the normality of the artificial BP measurements for individual volunteers, we conduct kurtosis and skewness tests [17], and determine whether the distribution of the artificial BP measurements that resample a normal curve is an approximately Gaussian distribution. We compute the artificial sample mean and variance as follows: ${\hat{\mu }}^{*}=\frac{1}{N}\sum_{b=1}^{N}{\hat{\theta }}_{b}^{S*}$ and ${\hat{\sigma }}^{*2}=\mathrm{var}\left({\hat{\Xi }}^{S*}\right)$. ${\hat{\Xi }}_{S*}=\left[{\hat{\theta }}_{1}^{S*},{\hat{\theta }}_{2}^{S*},\cdots ,{\hat{\theta }}_{N}^{S*}\right]$. We use Equations (11) and (12) to estimate the kurtosis and the standard error of the kurtosis.

(11) $$\mathrm{kurt}\left({\hat{\Xi }}_{S*}\right)=\left[\frac{E\left({\hat{\Xi }}_{S*}^{4}\right)-4{\hat{\mu }}^{*}E\left({\hat{\Xi }}_{S*}^{3}\right)+6{\hat{\mu }}^{*2}{\hat{\sigma }}^{*2}+3{\hat{\mu }}^{*4}}{{\hat{\sigma }}^{*4}}\right]$$

(12) $${\mathrm{se}}_{\mathrm{kurt}({\hat{\Xi }}_{S*})}={\left(\frac{24n{\left(n-1\right)}^{2}}{\left(n-2)(n-3)(n+5)(n+3\right)}\right)}^{\frac{1}{2}}$$

We use Equations (13) and (14) to estimate the skewness and the standard error of the skewness as follows:

(13) $$\mathrm{skew}\left({\hat{\Xi }}_{S*}\right)=\left[\frac{E\left({\hat{\Xi }}_{S*}^{3}\right)-3{\hat{\mu }}^{*}E\left({\hat{\Xi }}_{S*}^{2}\right)+2{\hat{\mu }}^{*3}}{{\hat{\sigma }}^{*3}}\right]$$

(14) $${\mathrm{se}}_{\mathrm{skew}({\hat{\Xi }}_{S*})}={\left(\frac{6n(n-1)}{\left(n-2)(n+1)(n+3\right)}\right)}^{\frac{1}{2}}$$

Therefore, we evaluate the normality based on the z-scores for kurtosis and skewness, as given by the following, respectively:

(15) $$z_{\mathrm{kurt}}=\frac{\mathrm{kurt}({\hat{\Xi }}_{S*})-0}{{\mathrm{se}}_{\mathrm{kurt}({\hat{\Xi }}_{S*})}}$$

(16) $$z_{\mathrm{skew}}=\frac{\mathrm{skew}({\hat{\Xi }}_{S*})-0}{{\mathrm{se}}_{\mathrm{skew}({\hat{\Xi }}_{S*})}}$$

where we set α to 0.05, and then z-scores to verify that we have an approximately Gaussian distribution. Based on this outcome, we consider further joint testing for kurtosis and skewness. With normality, the null hypotheses for these situations are expressed as follows:

(17) $$H_{0}:\mathrm{kurt}\left({\hat{\Xi }}_{S*}\right)=3\;\mathrm{and}\;\mathrm{skew}\left({\hat{\Xi }}_{S*}\right)=0.$$

### 4.3. Normality Test Using KS

The KS one-sample test is a technique for evaluating the correspondence between two sets of values [13]. The null hypothesis states that the artificial BP measurements have approximately Gaussian distributions. The alternative hypothesis is that the distributions of the artificial BP measurement are not approximately normal. We set the frequency level at α (=0.05). To conduct the KS test, we begin with a decision about the relative empirical distribution $\hat{F}\left({\hat{\Xi }}_{S*}\right)$, based on the observed artificial measurements. This test can be used to find a two-tailed probability *p* to determine if the artificial BP measurements are statistically similar or different by utilizing the point at which these two distributions exhibit the greatest divergence [17]. The *p* value less than the significant level indicates the distribution of artificial BP data that is not Gaussian. The *p* value greater than the significant level indicates the distribution of artificial BP data that is sufficiently Gaussian.

The KS test is based on the empirical distribution function [13] and uses a cumulative distribution function (CDF) $T\left(x\right)=P\left(X_{1}\leq x\right)$ (CDF) of the true underlying distribution of the data. Here, to simplify the following equations, we assumed that $x\equiv {\hat{\theta }}^{S*}$, $X_{1}\equiv {\hat{\theta }}_{1}^{S*}$ and $X\equiv {\hat{\Xi }}_{S*}$. We can define an empirical CDF in the following:

(18) $$T_{N}^{*}\left(x\right)=P_{N}\left(X\leq x\right)=N^{-1}\sum_{b=1}^{N}I_{\left[-\infty ,x\right]}\left(X_{b}\leq x\right)$$

where $I_{\left[-\infty ,x\right]}\left(X_{b}\leq x\right)$ represents an indicator equal to 1 if $\left(X_{b}\leq x\right)$ and equality to 0 otherwise, which determines the sample points ratio below level *x*. The law of large numbers is described such as

(19) $$N^{-1}\sum_{b=1}^{N}I_{\left[-\infty ,x\right]}\left(X_{b}\leq x\right)⟶E\left[I_{\left[-\infty ,x\right]}\left(X_{b}\leq x\right)\right]=P\left(X\leq x\right)=T\left(x\right)$$

The ratio of the BP data in the set [−∞,x] is close to the probability of this set.

**Theorem** **2.** *If T(x) continues, the distribution of the least upper bound is not rely on the unknown distribution P in the BP sample:*

(20) $$\|T_{N}^{*}{\left(x\right)-T\left(x\right)\|}_{\infty }⟶0$$

Therefore, this approximation remains uniformly across all x∈R, where ${\left\|\cdot \right\|}_{\infty }$ is $\sup_{x\in R}\left|\cdot \right|$. The result of Equation (16) demonstrates that the $T_{N}^{*}$ converges on the distribution of T in that the sup-norm of the difference has a probability of zero.

### 4.4. Independence Test Based on Rank

Next, we assume the *N* bivariate observations ${\hat{\Xi }}^{S*}=\left[{\hat{\theta }}_{1}^{S*},{\hat{\theta }}_{2}^{S*},\cdots ,{\hat{\theta }}_{N}^{S*}\right]$ and ${\hat{\Xi }}^{D*}=\left[{\hat{\theta }}_{1}^{D*},{\hat{\theta }}_{2}^{D*},\cdots ,{\hat{\theta }}_{N}^{D*}\right]$ for each individual subject (*i*) to be random resamples from the BP estimation results generated by the proposed approach, as shown in the algorithm. That is, the member of the $\left[{\hat{\Xi }}^{{\;}_{S*}},{\hat{\Xi }}^{{\;}_{D*}}\right]$ set were mutually independent and identically distributed in the bivariate population. We suppose that $T_{{\hat{\Xi }}^{{\;}_{S*}},{\hat{\Xi }}^{{\;}_{D*}}}$ are joint distribution functions for the bivariate population of the $\left[{\hat{\Xi }}^{{\;}_{S*}},{\hat{\Xi }}^{{\;}_{D*}}\right]$ set. Unlike other approaches, the Spearman rank-order correlation method deals with the relationship between two populations [13]. That is, this approach addresses how one population changes with respect to another. We obtain the z-value of a correlation coefficient (CORR) *r* and the CORR *r* for a large sample using the Spearman rank-order test [13]. As shown below, the null hypothesis indicates that there is no correlation (independence) between the artificial SBP and DBP measurements.

(21) $$H_{0}:\left[T_{{\hat{\Xi }}^{{\;}_{S*}},{\hat{\Xi }}^{{\;}_{D*}}}\left(\hat{\zeta },\hat{\eta }\right)\equiv T_{{\hat{\Xi }}^{{\;}_{S*}}}\left(\hat{\zeta }\right)T_{{\hat{\Xi }}^{{\;}_{D*}}}\left(\hat{\eta }\right),\;\forall \left(\hat{\zeta },\hat{\eta }\right)\right].$$

The level is the frequency set at α(=0.05), so there is a 95% confidence that the statistical difference of any observed BP measurement will be real and not due to chance. The asymptotic approximation utilizes *r*’s normality. When the null hypothesis is true, we obtain the expected value and variance of *r*. Thus, the expected value and variance of *r* are given by E(r)=0 and var(r)=1/N−1 under $H_{0}$, respectively.

## 5. Experimental Results and Comparison

We evaluated a protocol-based on BP measurement algorithm to ensure that the mean error (ME) is less than ±5 mmHg and that the error of standard deviation (SDE) is less than 8 mmHg [6]. For all BP measurements according to the British hypertension protocol (BHS) [18], we identified a % of the mean absolute error for three groups: 5 mmHg or less, 10 mmHg or less, and 15 mmHg or less. If 60% of the error measurements of BP algorithm is within 5 mmHg, 85% are within 10 mmHg, and 95% are within 15 mmHg, the algorithm can be classified as grade A.

### Statistical Analysis

Based on this configuration in Table 4, the mean errors in SBP and DBP values acquired from the deep learning method were compared with those acquired from the previous algorithms, as shown in Table 5. The error of the standard deviation obtained by the deep learning method was found to be 6.3 mmHg and 5.45 mmHg in the SBP and DBP. These results represent superior performance compared to previous algorithms. The results of the BHS protocol indicate that the deep learning method acquired better BP estimates when compared to the results of the previous methods as presented in Table 6.

According to the overall performance evaluation results, we can conclude that the proposed technique reduces the ME’s variance and increases the performance confidence level. As described in the introduction, the CLs of SBP and DBP acquired by deep learning method were narrower than those acquired by the previous methods as shown in Table 7. Although the CLs results obtained by the proposed method were wider than those obtained by [1], we noted differences of 2.0 mmHg and 1.6 mmHg in the SDEs of the CLs for the SBP and DBP, between the deep learning algorithm and the conventional method [1]. Thus, we can argue that the uncertainties of the SDEs were reduced when using the deep learning estimator.

**Figure 3:**
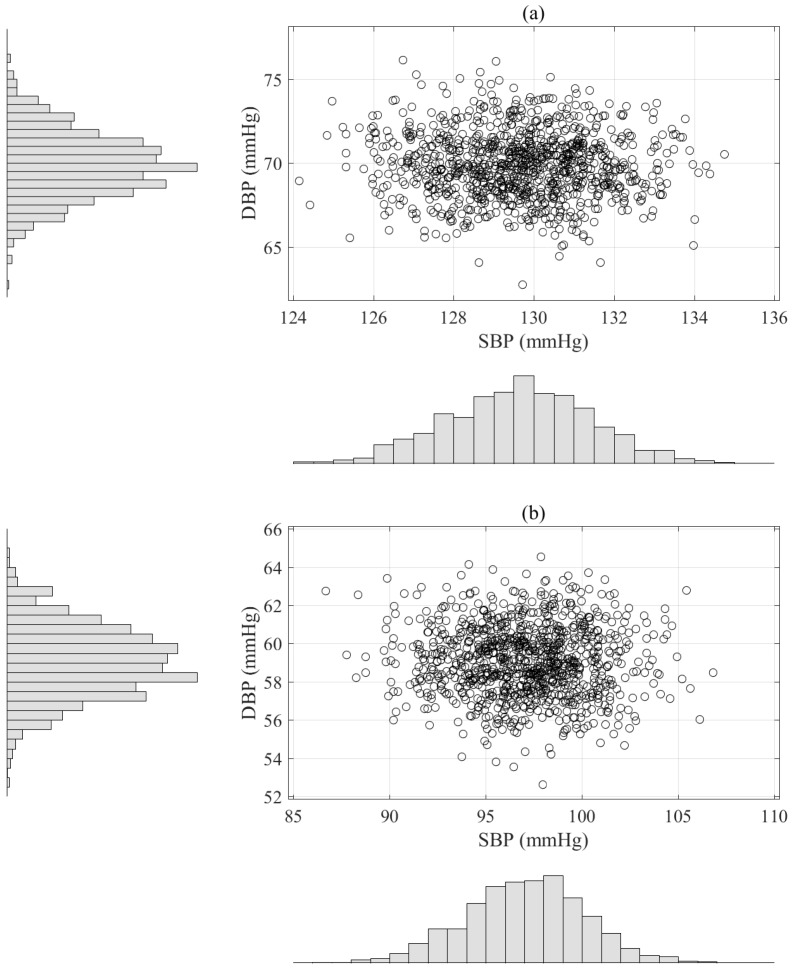
Scatter histograms of BP estimation based on the parametric bootstrap approach with replication numbers (*N* = 1000) using the results of the deep learning estimator, where the upper plot (**a**) is an example obtained from one subject and the bottom plot (**b**) is another example acquired from different subject.

To evaluate normality with respect to the individual BP measurements, we statistically analyzed the kurtosis, skewness, KS, correlation test results with their associated standard deviations (std) using the artificial BP measurements based on the results of the deep learning algorithm. Kurtosis is a measure of a population that identifies how flat or peaked it is in terms of a Gaussian distribution. The kurtosis for a Gaussian distribution is 3. Therefore, a kurtosis value greater than 3 indicates a heavy-tailed distribution and a kurtosis value of less than 3 indicate a light-tailed distribution. As shown in Figure 3 and Table 8, the kurtosis results for the SBP and DBP estimations were 2.99 and 3.01, which means that the distributions of the artificial BP measurements were almost normal. The skewness of a population can be represented as measure of its horizontal symmetry with respect to a Gaussian distribution. The skewness of a Gaussian distribution is 0 and symmetric data have a skewness of almost 0. The negative value is skewed to the left and the positive value to the right. We confirmed that the symmetric distributions of the artificial BP measurements were −0.01 and 0.01, respectively, for the SBP and DBP estimations, which indicates that they were very close to 0 for the number of bootstrap replications (*N* = 1000), as shown Figure 3 and Table 8.

**Figure 4:**
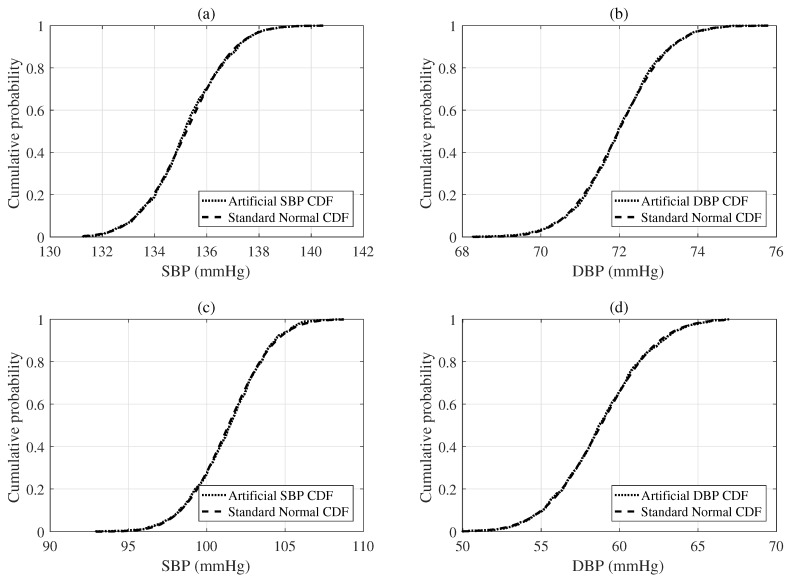
Cumulative distribution functions (CDFs) of selected artificial BP estimations obtained from the parametric bootstrap approach with replication numbers (*N* = 100) based on the results of the deep learning regression model. Note that the upper plot (**a**,**b**) are the examples obtained from one subject with 5 BP estimations and the bottom plot (**c**,**d**) are the examples acquired from another subject with 5 BP estimations.

We performed a KS test to verify the each distribution’s normality and showed that these were very similar to the normal distribution [19]. As the number of *N* bootstrap replications became large, the distribution of the artificial BP measurement was close to a Gaussian distribution. We confirmed the distribution of the artificial BP measurement to be a Gaussian distribution based on the test results, which represent the cumulative distribution function (CDF) of the artificial BP measurements versus the theoretical CDF of a Gaussian distribution, as shown in Figure 4.

Moreover, we confirmed that the hypothesis results *h* for the mean artificial BP measurements were 0 for the SBP and DBP estimations. These results indicate that we accepted the null hypothesis at the 0.05 significance level. In addition, we could not reject the null hypothesis because the ks (=0.02) values were less than the critical values cv (=0.04). Also, our *p* (=0.78) and (=0.79) KS test values were greater than the α (=0.05) significance level for SBP and DBP, as shown in Table 8. Therefore, we accepted the normality of the distribution of the artificial BP measurements for individual subjects. In addition, we obtained CORR $r_{s}$ (=0.01) and $r_{d}$ (=0.01) values based on the asymptotic normality between the artificial SBP and DBP measurements and acquired variances of 0.0009 and 0.0009 for $r_{s}$ and $r_{d}$ using the independence based on rank as described the last column in Table 7, respectively. Therefore, these results are clearly close to the expected values and variances. Thus, we did not reject $H_{0}$ (independence) at the α=0.05 level.

## 6. Conclusions

The main contribution of this paper was our verification of the normality of the BP data, using only five samples, using various statistical methodologies for individual subjects. We clearly determined the independence of the artificial SBP and DBP estimations using the deep learning model based on the distribution-free test of rank. The proposed methodology also provides accurate BP estimations and reduces uncertainties such as the CLs and SDEs, as determined by the deep learning regression estimator. In the near future, we will pursue additional non-normally testing using a new subject populations.

## Figures and Tables

**Table 1:** The statistical information about eighty five volunteers.

Statistical Information	Value
Age (Male)	12 to 80
Age (Female)	17 to 65
Arm size	25 (cm) to 42 (cm)
Wrist size	13.5 (cm) to 23 (cm)
Deflation rate	3.0 (mmHg/s)
Male	48 of 85 (56.5%)
Female	37 of 85 (43.5%)

**Table 2:** The hypothesis *h* values are represented from the artificial feature using the bootstrap approach with the number of replications (*N* = 100), where *p* is the *p*-value, ks denotes the statistical value of the Kolmogorov-Smirnov test, and cv is the threshold value. Other features show similar results.

Feature/Test Values	*h*	*p*	*ks*	*cv*
MAP	0	0.900	0.056	0.134
AR	0	0.904	0.055	0.134
AE	0	0.885	0.057	0.134
EL	0	0.716	0.068	0.134
MA	0	0.599	0.075	0.134
$\sigma_{1}$	0	0.996	0.040	0.134
$\sigma_{2}$	0	0.949	0.051	0.134
MAPL	0	0.969	0.048	0.134

**Table 3:** An exemplary result (second volunteer) representing an validation between the normalized artificial features and normalized original features for consistency and convergence [12].

Features	$\hat{\theta }$	${\hat{\theta }}^{*}$	${\mathrm{CL}}_{L}^{*}$	${\mathrm{CL}}_{U}^{*}$	$\beta ({\hat{\theta }}^{*}\left(\cdot \right))$	$\hat{S_{e}}$	${\hat{S_{e}}}^{*}$
TSBP	95.4 (3.4)	95.5(1.6)	92.3	98.9	0.1	3.36	1.59
TDBP	64.8 (3.6)	64.7(1.7)	61.3	68.6	−0.1	3.63	1.70
MAP	0.3703 (0.07)	0.3707(0.03)	0.031	0.045	0.0004	0.07	0.03
AR	0.4960 (0.05)	0.4930 (0.03)	0.440	0.547	−0.003	0.047	0.025
AE	0.0670 (0.007)	0.0680 (0.003)	0.061	0.074	0.001	0.008	0.003
EL	0.0480 (0.023)	0.0478 (0.010)	0.029	0.068	−0.0002	0.023	0.01

**Table 4:** Parameters, [8,9] of the deep learning algorithm.

Number of the Hidden Unit in Three Layers:	[(11,(32),(32),(32),4)]
Size of input vector **X**	11
Size of output vector **Y**	2
Number of sample over each pseudo feature	100
Number of sample over each original feature	5
Number of hidden layers	3
Number of hidden unit on the layers	16 to 256
Number of ensemble	50
Learning rate for weight	0.001
Learning rate for biases of visible units	0.01
Learning rate for biases of hidden units	0.01
Momentum rate	0.9
Activation type	sigmoid type function
Maximum epoch in the pre-training	200
Maximum epoch in the fine-tuning	200
Initial weights and biases	randomly between (−1, 1)

**Table 5:** Mean error (ME) and standard deviation of error (SDE) relative to the reference auscultatory method obtained with the conventional maximum amplitude algorithm (MAA) [1], neural networks (NN) [4], support vector regression (SVR) [14] and deep neural network’s (DNN) regression model [10], where the results are the average values for our test data.

mmHg	MAA		NN		SVR		DNN	
Test	SBP	DBP	SBP	DBP	SBP	DBP	SBP	DBP
ME	0.07	−0.89	0.25	−0.22	−0.51	0.17	0.36	−0.61
SDE	9.28	7.76	7.48	6.80	7.20	6.18	6.30	5.45

**Table 6:** Grading of the proposed algorithm based on the BHS standard using the results of MAA, NN, SVR, and DNN on (5×85=425) measurements.

	SBP			DBP			Standard (SBP/DBP)
Tests	Absolute Difference (%)			Absolute Difference (%)			BHS
	≤5 mmHg	≤10 mmHg	≤15 mmHg	≤5 mmHg	≤10 mmHg	≤15 mmHg	Grade
MAA	47.06	85.88	96.47	56.47	88.24	97.65	C/B
NN	53.88	85.65	95.53	66.12	94.12	98.82	B/A
SVR	62.59	86.12	95.53	74.12	93.65	96.94	A/A
DNN	69.18	88.71	97.18	76.24	93.17	98.12	A/A

**Table 7:** Summary of the CL of the SBP and DBP for nurse measurements, the proposed DNN regression model and the conventional methods, *n* (=85) is the number of subjects and SDE is a standard deviation of error; L and U are the lower and upper limits, respectively.

BP (mmHg)	SBP (SDE)	DBP (SDE)	SBP L (SDE)	SBP U (SDE)	DBP L (SDE)	DBP U (SDE)
	95%CI	95%CI				
${\mathrm{MAA}}_{\mathrm{ST}}$ [1]	13.2 (8.0)	9.4 (5.8)	106.7 (14.3)	120.2 (16.5)	62.4 (10.4)	71.7 (11.0)
${\mathrm{MAA}}_{\mathrm{GUM}}$ [1]	13.9 (7.9)	10.0 (5.4)	106.4 (14.3)	120.5 (16.4)	62.0 (10.4)	72.1 (10.9)
${\mathrm{PMAE}}_{\mathrm{NPB}}$ [1]	2.8 (3.3)	1.7 (2.4)	112.4 (13.9)	115.7 (14.1)	66.7 (10.5)	68.2 (9.9)
${\mathrm{DNN}}_{\mathrm{Boot}}$	5.5 (1.3)	4.2 (0.8)	107.4 (12.7)	113.0 (12.6)	64.5 (8.3)	68.6 (8.4)

**Table 8:** Statistical results such as skewness, kurtosis, Kolmogorov-Smirnov (KS), Spearman’s correlation between the SBP and DBP estimations, where the results are the average values for our test data.

Tests	KS Test				Normality Test		
α (=0.05)	h (std)	p (std)	ks (std)	cv (std)	Kurtosis (std)	Skewness (std)	corr (std)
SBP	0 (0)	0.78 (0.2)	0.02 (0.00)	0.04 (0.00)	2.99 (0.14)	−0.01 (0.08)	0.01 (0.03)
DBP	0 (0)	0.79 (0.2)	0.02 (0.01)	0.04 (0.00)	3.01 (0.16)	−0.01 (0.07)	0.01 (0.03)
